# “My Goal Is to Lose 2.923 kg!”—Efficacy of Precise Versus Round Goals for Body Weight Reduction

**DOI:** 10.3389/fpsyg.2022.793962

**Published:** 2022-02-07

**Authors:** Marie-Lena Frech, Malte Friese, David D. Loschelder

**Affiliations:** ^1^Institute of Management & Organization, Leuphana University, Lueneburg, Germany; ^2^Department of Psychology, Saarland University, Saarbruecken, Germany

**Keywords:** goal setting, numeric precision, field experiments, weight loss, health

## Abstract

Overweight individuals often struggle to lose weight. While previous studies established goal setting as an effective strategy for weight loss, little is known about the effects of numeric goal precision. The present research investigated whether and how the precision of weight loss goals—the number of trailing zeros—impacts a goal’s effectiveness. In two preregistered, longitudinal experiments, we contrasted competing predictions as to whether precise (e.g., 2.923 kg) or round (e.g., 3.000 kg) goals are more effective compared to a waiting control condition. In Experiment 1 (*N* = 121), participants in the two goal conditions lost more weight compared to the control condition—an effect that was mainly driven by precise (rather than round) goals. In Experiment 2 (*N* = 150), we sought to replicate this effect but found no significant weight loss differences. An individual participant data (IPD) meta-analysis across both experiments revealed that (a) the goal groups jointly lost more weight than the waiting control group and (b) the precise and round goal groups did not differ in weight loss success. An IPD-based multiple mediation analysis showed that healthier eating, but not physical exercise accounted for goal-setting-induced weight loss. We discuss possible explanations for the null findings in Experiment 2 and highlight directions for future research.

## Introduction

According to the World Health Organization (WHO), the worldwide obesity rate has nearly tripled since 1980 and is still rising (World Health Organization, 2020). Increased caloric intake and poor physical activity habits are linked to obesity and represent a major risk factor for diseases (Shilts et al., 2004). To counteract this societal trend, goal setting may potentially serve as an effective strategy for dietary behavior change (Shilts et al., 2004). Recent research on the consequences of precise versus round numbers in the context of judgments (Jerez-Fernandez et al., 2014), decision-making (Schultze and Loschelder, 2020), estimations (Frech et al., 2020), and negotiations (Mason et al., 2013; Loschelder et al., 2016) leads to competing theoretical predictions about the relative effectiveness of precise versus round weight loss goals. One line of research and theorizing suggests that precise weight loss goals (e.g., 2.923 kg) should be more effective compared to round goals (e.g., 3.000 kg; Janiszewski and Uy, 2008); another line of research and theorizing suggests the opposite (Pope and Simonsohn, 2011). The present research seeks to bridge the literature on weight loss efficacy and the literature on numeric precision effects. Specifically, in two preregistered, longitudinal field experiments, we examined whether setting a precise or round weight loss goal is more promising for weight loss success.

### Goal Setting in the Health Context

Goal setting theory (Locke et al., 1981; Locke and Latham, 1990) states that setting and subsequently monitoring a goal substantively helps people to meet their aspirations. A goal is defined as what the individual consciously seeks to accomplish (Lunenburg, 2011). Abundant evidence from diverse domains, such as the workplace (Locke, 1968), sports (Kingston and Wilson, 2008), consumer behavior (Breedvelt et al., 2020), and the health context (Strecher et al., 1995; Cullen et al., 2001) indicates that goal setting is a powerful technique for motivating individuals to engage in action and to reduce the discrepancy between the current status quo and a desired end state (Locke and Latham, 2006). Several literature reviews examined the influence of goal setting strategies on dietary and physical activity behavior changes (Shilts et al., 2004; Pearson, 2012) and found positive effects of goal setting on decreased fat intake, increased fiber consumption, and higher exercise adherence, among others.

Goal specificity is one aspect that is particularly helpful to make goal setting effective for behavior change (Bandura and Simon, 1977; Kyllo and Landers, 1995; Shilts et al., 2004; Bodenheimer and Handley, 2009; Pearson, 2012). Specific goals provide a concrete desired end state that allows to quantify progress. In contrast to specific goals, unspecific goals are characterized by ambiguity or diffuseness in the required level of performance (“do your best”; Locke and Latham, 2002). Previous research showed that individuals who set themselves specific goals for their daily caloric intake (e.g., 2,000 calories per day) were more successful in weight loss than individuals who set themselves vague, non-quantitative goals (e.g., “reduce caloric intake”; Bandura and Simon, 1977). Further, overweight women who wrote down specific weekly goals for changes in weight and eating behavior were more successful in a 7-week weight loss period than women who solely had the general goal to lose weight (Chapman and Jeffrey, 1978).

### Goal Setting and Numeric Precision

Goal specificity distinguishes quantified, specific targets from unspecific, “do your best” goals. The numeric precision approach goes one step further and additionally distinguishes specific goals numerically as “round” (e.g., 2,000 calories) versus “precise” (e.g., 1,985 calories)—frequently defined as a function of the number of trailing zeros (e.g., Mason et al., 2013; Frech et al., 2020). According to this definition, precise numbers are those with fewer trailing zeros in it (Mason et al., 2013; Loschelder et al., 2016). Thus, a goal of 2.923 kg is numerically more precise compared to a goal of 3.000 kg, since the round number entails more zeros compared to the precise number. In the present research, we aimed to investigate numeric precision as a novel goal characteristic of specific goals.

Recent precision research in areas such as judgment and decision-making, social cognition, and negotiations suggests that precise numbers—those with fewer trailing zeros—affect estimations, judgments, and interpersonal processes markedly differently than round numbers (e.g., Janiszewski and Uy, 2008; Wadhwa and Zhang, 2015; Loschelder et al., 2016). Here, we applied and extended this emerging theorizing and empirical knowledge to the context of weight loss goals and goal attainment. This is a pertinent research question not only for the applied purposes to identify means to maximize goal attainment in the domain of weight reduction. It is also theoretically relevant because the *a priori* predictions are not straightforward: Two different lines of reasoning result in opposing predictions as to whether precise or round weight loss goals are more effective.

#### Precise Goals Might Outperform Round Goals

The first theorizing—the so-called “scale-granularity account”—is derived from the anchoring literature (Janiszewski and Uy, 2008). Anchoring is one of the most robust phenomena in human decision-making and constitutes the assimilation of a numeric estimate to a previously considered standard (Tversky and Kahneman, 1974; Mussweiler and Strack, 2000). Precise numbers have been shown to function as more potent anchors than round ones (Mason et al., 2013; Loschelder et al., 2014). This means that responses of people confronted with a precise number (the anchor) were more strongly influenced by the anchor than responses of people confronted with a round number. For example, individuals who were estimating the value of an object based on a precise price assimilated their estimate more towards the anchor compared to individuals who based their estimate on a round number (Frech et al., 2020).

Different mechanisms have been suggested to account for the stronger anchoring potency of precise numbers. One of the most prominent ones is described by the “scale-granularity account.” The account builds on the assumption that people adjust away from anchors in serial steps (Epley and Gilovich, 2001, 2006) and that precise numbers influence the resolution of individuals’ mental adjustment scale (Janiszewski and Uy, 2008): Precise numbers (e.g., €5,124) induce a finer-grained mental scale that leads people to adjust away from the anchor in smaller individual steps, such as €25 or €50. In contrast, a round number, such as €5,100, induces a more coarse-grained scale, leading people to adjust in larger steps, such as €100 or €200. The same number of steps on a coarse-grained scale will move people farther away from an anchor than if they adjust in smaller steps on a finer scale (see Frech et al., 2020, for recent evidence supporting this account).

Based on this scale-granularity theorizing, one could assume that precise weight loss goals (e.g., 2.923 kg) also induce a finer-grained mental scale when individuals work towards a self-set precise compared to a round weight loss goal (e.g., 3.000 kg). Consequently, individuals would strive to move in smaller, possibly more achievable steps towards their goal, say, 200 g per week or 25 g per day. In comparison, round weight loss goals (3.000 kg) might induce a coarse-grained scale (Janiszewski and Uy, 2008) of, say, striving to lose 500 g or even 1 kg per week. This might result in seemingly insurmountable intermediate steps that could result in a lack of motivation and even goal frustration. Thus, according to the scale-granularity account, one would predict that precise (rather than round) goals increase weight loss motivation—many small, achievable steps on a regular basis—and hence result in more success in weight loss.

#### Round Goals Might Outperform Precise Goals

A different theoretical account renders the opposite prediction equally reasonable. This “reference point account” indicates that, in a performance context, round goals can function as a cognitive reference point. Individuals judge their respective outcomes in comparison with this reference point, for instance, while taking the Scholastic Aptitude Test (SAT, “I really want to score at least 1,200 points”; Pope and Simonsohn, 2011). A reference point then qualifies one’s outcome into a subjective gain or loss depending on whether the outcome is above or below the salient reference point (Allen et al., 2017). As a result, people may experience greater motivation to exert more effort when they are just short of meeting their reference point (Heath et al., 1999). The idea that motivation increases with proximity to the goal is also in line with the goal-gradient hypothesis (Hull, 1932; Kivetz et al., 2006). Importantly, according to the reference point account, people prefer round over precise reference points because round numbers as cognitive reference points occur more naturally (Pope and Simonsohn, 2011). Further, round numbers as goals are evaluated even more favorably because they render strong associations with completeness and individuals exert extra effort to complete their goals at round numbers (Pope and Simonsohn, 2011; Gunasti and Ozcan, 2016).

Empirical evidence from diverse domains supports this reference point account. For instance, marathon finishing times are bunching just below round numbers (e.g., a 4-h marathon), indicating that these round numbers serve as meaningful reference points and that runners are more likely to speed up to finish just shy of the round 4-h reference point (i.e., the “round time”; Allen et al., 2017). Similarly, baseball players whose seasonal batting average is just below a round number (e.g., 0.298) exert stronger efforts in their last games to finish just above the round number (e.g., 0.300; Pope and Simonsohn, 2011). Finally, students scoring just short of a round number in their SAT are more likely to retake the test than students scoring farther away from a round number (Pope and Simonsohn, 2011). In all, this line of research and reasoning suggests that round goals—as salient reference points—are perceived as more rewarding and that individuals are more motivated to work towards round compared to precise goals.

In sum, both accounts should have a motivational effect on the weight loss process. The scale-granularity account suggests smaller incremental, achievable steps towards one’s precise goal to be more motivating and thus more conducive to foster weight loss (Janiszewski and Uy, 2008; Frech et al., 2020). In contrast, the reference point account would predict that round goals result in greater weight loss success because individuals are motivated to perform just above their round goal rather than to fall just short of this goal (Pope and Simonsohn, 2011).

## Contributions and Overview

The present research aimed to empirically contrast these competing hypotheses of the scale-granularity account and the reference point account and to expand the goal setting literature by casting light on a novel goal characteristic: the numeric precision of self-set goals for weight loss. In two preregistered, longitudinal field experiments, we examined the influence of precise (e.g., 2.923 kg) versus round (e.g., 3.000 kg) goals on weight loss success. Both goal groups were compared to a no-goal waiting control condition.

## Experiment 1

In Experiment 1, individuals who sought to lose weight took part in a longitudinal field experiment for the duration of 6 weeks. The precise goal group was asked to choose a precise number of kilograms and grams that they wanted to lose (e.g., 2.923 kg) while the round goal group was asked to choose a round number of kilograms that they wanted to lose (e.g., 3.000 kg). The no-goal waiting control condition was informed that due to organizational reasons they would start the weight loss program 6 weeks later. Weight measures were assessed before and 6 weeks after the goal setting manipulation. Based on goal setting theory, we predicted that both goal groups (precise and round) would lose more weight in 6 weeks than the waiting control group (Hypothesis 1). Moreover, we explored whether either precise (Hypothesis 2a) or round goals (Hypothesis 2b) would be particularly effective for goal achievement.

### Methods

#### Preregistration

The preregistration to this study can be found at https://osf.io/mdkhx/. Deviations from the preregistration are explicitly noted in the manuscript. Analyses marked as “preregistered” correspond to all confirmatory analyses that were included in the preregistration. Analyses marked with “non-preregistered” correspond to explorative analyses that were not included in our preregistration.

#### Design and Participants

The experiment followed a 1 × 3 between-subjects design (precise goal vs. round goal vs. no-goal control condition). Sample size was determined *a priori* using G*Power (Faul et al., 2007). Because—to our knowledge—no prior studies had examined the influence of goal precision on weight loss, we assumed a conventionally moderate effect size of *f* = 0.25 for our power analyses. The other parameters were *α* = 0.05, statistical power of 1−*β* = 0.80 and correlation among repeated measures of *r* = 0.7. For a 3 × 2 (group × time) mixed ANOVA with repeated-measures for the time factor, this led to a minimum sample size of 135 participants (*n* = 45 per condition). We wish to note transparently that the preregistration contains an error in that we powered for a repeated-measures ANOVA although we preregistered weight difference (time 1 − time 2) as our main dependent variable, which results in a 1 × 3 between-subjects ANOVA. The G*Power analysis for the one-way ANOVA leads to a sample size of 159 participants. With the sample size of the repeated-measures ANOVA (*N* = 135), we thus only had 73% power instead of 80%. For reasons of simplicity, we report the 1 × 3 ANOVA in the subsequent result section. Importantly, this one-factorial ANOVA produces a main effect that is equivalent to the interaction effect in the 3 × 2 repeated-measures ANOVA.

Participants were recruited at the campus of the Leuphana University Lueneburg, via flyers in the surrounding area, and at the “Weight Watcher Center” in Lueneburg. All participants reported that they wanted to lose weight. Data were not analyzed prior to termination of data collection (i.e., after a total of 8 weeks and once all signed-up participants had participated). We recruited all 156 participants that signed up for the study within the first 2 weeks of data collection. Excess participants beyond the minimum of 135 were retained in the data sample, assuming that there might be drop-outs. Ten participants whose body mass index (BMI) was below 20, could not take part in the study (see predefined criteria in the preregistration). Nineteen (out of the total of 156) participants did not show up at their second appointment and therefore had to be excluded from the sample (12.2% dropout). Three subjects were excluded because the online software failed to record their data, and one person from the round group was excluded because she did not follow the instruction to set herself a goal. Two participants were excluded because their weight loss score exceeded more than the preregistered ±2.5 *SD* from the respective condition mean. This resulted in a total sample size of *N* = 121 (*M*_age_ = 24.77, *SD* = 9.13, 18–66 years, 102 females).

#### Procedure

At their first appointment, all participants were measured (height) and weighed and then answered a computer-based questionnaire via the software *SoSciSurvey* (https://www.soscisurvey.de/de/about; see questionnaire appointment 1 below). Upon completion of the questionnaire that also contained the experimental manipulation, participants were thanked and reminded of their appointment 6 weeks later. To minimize dropout, all participants were also reminded of their participation in the study via a text message (or email) after 2 and 4 weeks into the study. At the second appointment (6 weeks after the first appointment), participants were again welcomed and weighed and filled in a questionnaire to assess their eating and physical exercise behavior during the previous 6 weeks (see questionnaire appointment 2 below). Finally, participants were debriefed (especially participants in the no-goal control group were informed that they were assigned to the control group), remunerated (€7 or 1.5 h course credit), and thanked for their participation.

#### Experimental Manipulation

Participants were randomly assigned to one of three experimental conditions prior to their arrival at the laboratory (precise goal vs. round goal vs. waiting control condition). In the two goal groups, participants were asked to choose a precise or round weight loss goal. In the precise goal condition, participants read: “Goals are particularly motivating and effective, when they are precisely formulated—that means precise to the gram. Examples are ‘I want to lose 1 kilogram and 875 grams’ compared to ‘I want to lose about 2 kilograms’ […]. In order for your weight reduction to be as successful as possible, please consider a precise goal before starting the study, that is, how many kilograms and grams you would like to lose.” Subsequently, participants were asked to enter their precise self-set goal. In the round goal condition, participants read: “Goals are particularly motivating and effective, when they are round. Examples are ‘I want to lose 2 kilograms’ or ‘I want to lose 4 kilograms’ […]. In order for your weight reduction to be as successful as possible, please consider a round goal before starting the study, that is, how many kilograms you would like to lose.” Again, participants were then asked to enter their round self-set goal. In the waiting control condition, participants seeking to lose weight learned that they would start with the experiment 6 weeks later and that, for now, we would only assess their weight at the beginning and at the end of these 6 weeks. Both goal groups wrote down their goal on five stickers and were asked to place these stickers prominently in their respective apartment (e.g., on the TV, mirror, and fridge). All participants were contacted via text message or email after 2 and 4 weeks. Participants in the control condition were reminded of their participation in the study. Participants in both goal groups were asked to respond to the message with their self-set weight loss goal.

#### Dependent Measures

##### Body Measurements

Participants’ height was determined with a measuring tape. We assessed participants’ weight (in kg), their BMI, body fat (%), muscle mass (%), and visceral fat (%) with a medical body fat monitor (Omron BF-511). At each of the two laboratory appointments, body measurements were taken twice in a row (*r*s > 0.95, *p*s < 0.001) and averaged into a single score to reduce measurement error.

##### Questionnaire Appointment 1

We assessed participants’ demographic data (e.g., age and gender) and a number of possible moderating variables that could explain and alter a potential effect of goal precision on weight loss. For example, we assessed participants’ trait self-control (Tangney et al., 2004) and weight efficacy (Clark et al., 1991). For a full list of measures and verbatim items, please refer to the study’s OSF project.

##### Questionnaire Appointment 2

At the second appointment, we assessed a number of variables that might have influenced participants’ success in weight loss. For example, we asked participants about their eating behavior (e.g., “In the last 6 weeks, I refrained from eating sweets”; scale ranged from *1 = completely disagree* to *7 = completely agree*) and their physical exercise behavior (“How often were you physically active during the 6 weeks weight loss phase?” scale ranged from *1 = not at all* to *8 = daily*) in order to examine how precise versus round goals helped participants to reduce their weight relative to a waiting control condition (see project on the OSF for a full list of measures and verbatim items).

#### Main Dependent Variables

Participants’ weight loss (i.e., difference of weight_T1_ − weight_T2_) and the goal discrepancy (difference of desired weight loss minus factual weight loss; in %) served as our key dependent variables. Please refer to the supplementary online material (SOM) for a number of additional analyses.^1^

### Results

#### Preliminary Analyses (Preregistered)

The three experimental conditions did not differ in terms of starting weight (*M*_precise_ = 69.81 kg, *SD* = 11.66; *M*_round_ = 71.42 kg, *SD* = 11.85; *M*_control_ = 67.31 kg, *SD* = 13.33), *F*(2, 118) = 1.05, *p* = 0.354, *η_p_*^2^ = 0.02. The ambition of the self-set goals also did not differ between the precise and the round group (*M*_precise_ = 3.07, *SD* = 1.22; *M*_round_ = 3.47, *SD* = 1.39), *t*(78) = 1.34, *p* = 0.183, *d* = 0.31; please refer to the SOM for additional preliminary analyses. A Shapiro–Wilk test for our main dependent variables, weight loss and goal discrepancy, showed that both variables were normally distributed (*W*_weight loss_ = 0.98, *p* = 0.299; *W*_goal discrepancy_ = 0.99, *p* = 0.471).

#### Goal Setting Efficacy for Weight Loss (Preregistered)

**Figure 1:**
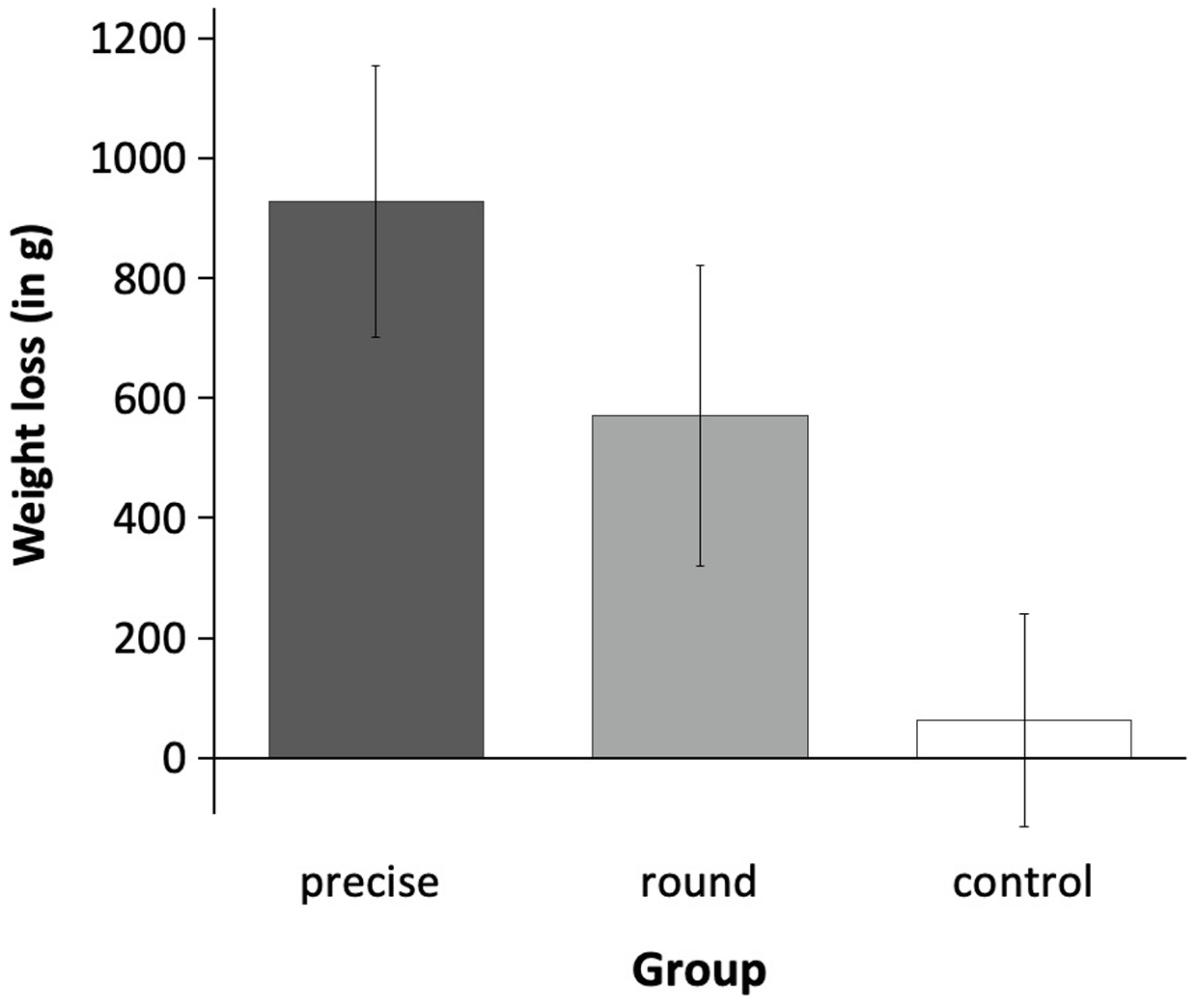
Weight loss in Experiment 1. The three experimental conditions (precise goal vs. round goal vs. control) differed significantly in weight loss. Contrast analyses showed that—considered jointly—both goal groups lost significantly more weight than the control group. This significant contrast was driven in particular by the precise goal condition. Error bars represent ±1 SEM.

To examine whether the three experimental conditions differed in weight loss, we conducted an ANOVA with weight loss (weight_T1_ − weight_T2_) as the dependent variable. As Figure 1 illustrates, the three groups differed significantly in weight loss, *F*(2, 118) = 4.26, *p* = 0.016, *η_p_*^2^ = 0.07. In line with H1, planned *post hoc* contrast analyses (−2 1 1) showed that when considered jointly, the goal conditions (*M*_precise_ = 0.93 kg, *SD* = 1.57; *M*_round_ = 0.57 kg, *SD* = 1.41) lost significantly more weight than the control condition (*M*_control_ = 0.06 kg, *SD* = 1.14), *t*(118) = −2.54, *p* = 0.012, *d* = 0.47.

To test whether participants setting precise (H2a) or round goals (H2b) differed in their weight loss, we calculated simple contrasts. The results showed, that descriptively, the precise group (*M* = 0.93 kg, *SD* = 1.57) lost more weight than the round group (*M* = 0.57 kg, *SD* = 1.41). However, this difference was not significant, *t*(118) = −1.12, *p* = 0.264, *d* = 0.24. Additionally, we explored whether the two goal groups differed from the control condition (non-preregistered). The difference between the precise goal group (*M* = 0.93 kg, *SD* = 1.57) and the control group (*M* = 0.06 kg, *SD* = 1.14) was significant, *t*(118) = −2.92, *p* = 0.004, *d* = 0.63, whereas the difference between the round group (*M* = 0.57 kg, *SD* = 1.41) and the control group (*M* = 0.06 kg, *SD* = 1.14) did not reach significance, *t*(118) = −1.54, *p* = 0.126, *d* = 0.39. The slightly greater weight loss of the precise goal condition, followed by the round goal condition and the control condition, was reflected in a significant linear contrast (+1 = precise, 0 = round, −1 = control), *t*(118) = 2.92, *p* = 0.004, *d* = 0.53 (see Figure 1).

#### Goal Setting Efficacy for Weight Loss (Non-preregistered)

We also conducted one-sample *t*-test to check whether the weight loss in each condition differed significantly from zero. The weight loss in the control condition did not differ significantly from zero [*t*(40) = 0.36, *p* = 0.723, *d* = 0.06], while the precise [*t*(47) = 4.10, *p* < 0.001, *d* = 0.59] and the round condition [*t*(31) = 2.28, *p* = 0.030, *d* = 0.40] differed significantly from zero.

#### Precise Versus Round Goal-Efficacy (Preregistered)

We also examined how much participants’ actual weight loss differed from their desired weight loss (i.e., goal discrepancy). On average, individuals in the precise group achieved their goal to a percentage of 39.55%, while the round group achieved their goal to a percentage of 26.92%. This difference was not significant, *t*(78) = −1.38, *p* = 0.173, *d* = 0.31.

#### Robustness Checks (Non-preregistered)

We also calculated separate ANCOVAs that controlled for age, gender, Weight Watcher versus student participants, and a number of trait personality measures, such as trait self-control, weight efficacy, self-efficacy for physical exercise behavior, and restraint eating. These ANCOVAs consistently showed the same condition main effect; all *F*s > 3.21, *p*s < 0.025, *η_p_*^2^s > 0.05 (please refer to the SOM for details).

#### Eating Behavior and Physical Exercise Behavior (Non-preregistered)

Finally, we tested whether the three groups differed in eating behavior and physical exercise behavior. The results showed that the three groups differed significantly in eating behavior, *F*(2, 118) = 5.23, *p* = 0.007, *η_p_*^2^ = 0.08. Simple contrast analyses revealed that the precise goal group (*M* = 4.44, *SD* = 0.97) reported eating significantly healthier than the control group (*M* = 3.77, *SD* = 1.28) during the 6 weeks of the experiment, *t*(118) = 2.60, *p* = 0.011, *d* = 0.47. The difference between the round goal group (*M* = 4.61, *SD* = 1.45) and the control group (*M* = 3.77, *SD* = 1.28) was also significant, *t*(118) = 2.95, *p* = 0.004, *d* = 0.54, indicating that the round group reported eating healthier than the control group. The difference between the two goal groups (*M*_precise_ = 4.44, *SD* = 0.97; *M*_round_ = 4.61, *SD* = 1.45) was not significant, *t*(118) = 0.625, *p* = 0.533, *d* = 0.11.

There was no significant difference between the three groups regarding physical exercise behavior during the 6 weeks of the experiment, *F*(2, 118) = 0.97, *p* = 0.383, *η_p_*^2^ = 0.02.

### Discussion

Corroborating previous research, Experiment 1 showed that goal setting was an effective strategy to lose weight. Considered jointly, both goal conditions lost more weight than a waiting control group during the 6 weeks of this study. The beneficial results for goal setting seemed to be driven predominantly by the precise goal group, as indicated by only the precise condition lost significantly more weight than the control group. Descriptively, the precise goal group lost more weight than the round goal group; however, this difference was not significant—possibly because the 6-week duration of weight loss was not sufficient to find the predicted differences. The results also revealed that both the precise and the round goal group reported eating significantly healthier during the 6 weeks of the experiment compared to the control group.

## Experiment 2

### Methods

#### Preregistration

The preregistration for this study can be found at https://osf.io/aj78d/. Deviations from the preregistration are explicitly noted in the manuscript.

#### Design and Participants

Again, the experiment followed a 1 × 3 between-subjects design (precise goal vs. round goal vs. no-goal control condition). The same G*Power analysis as in Experiment 1 again yielded a minimum sample size of 135 participants (*n* = 45 per condition). The recruitment of participants took place at the Leuphana University Lueneburg and the Saarland University, simultaneously. At both universities, participants who wanted to lose weight were recruited through the respective online research participation system, on campus, and via flyers in the surrounding areas. Participants who took part in Experiment 1 were not allowed to participate in this experiment. Participants received course credit (2 credit hours) or money (€10) for participation. Data were not analyzed prior to termination of data collection (i.e., after a total of 11 weeks and once all signed-up participants had participated).

We recruited a total of 191 participants (131 subjects in Lueneburg, 60 subjects in Saarbruecken) who were measured and weighed at their first appointment. Thirty-four participants did not show up to their second appointment and therefore had to be excluded from the sample (17.8% dropout). One person was excluded because the software failed to record her data. One subject from the round condition was excluded for not following the instruction to set herself a goal, and one person was excluded from the precise group because she set herself a round goal (see preregistered exclusion criteria). Four participants were excluded because their weight loss score exceeded more than ±2.5 *SD* from the respective condition mean (see the preregistered criteria). This resulted in a total sample size of *N* = 150 (*M*_age_ = 22.95, *SD* = 3.54, 18–45 years, 114 females).

#### Procedure

The procedure was similar to Experiment 1 except that the second appointment occurred after 8 weeks instead of 6 weeks. All participants were reminded of their participation in the study via text message (or email) after 3 and after 5 1/2 weeks. In addition, participants in the goal conditions received a text message after 2 days in which they were reminded to place the stickers with their goals at prominent places in their respective apartments.

#### Experimental Manipulation

The experimental manipulation was identical to the one in Experiment 1.

#### Dependent Measures

##### Body Measurements

Body measurements were identical to Experiment 1. At each of their two laboratory appointments, participants’ body measurements were again taken twice in a row (*r*s > 0.97, *p*s < 0.001) and averaged into a single score.

##### Questionnaire Appointment 1

The questionnaire for the first appointment was identical to the one used in Experiment 1.

##### Questionnaire Appointment 2

Similar to Experiment 1, we assessed participants’ physical exercise behavior and their eating behavior during the 8 weeks of weight loss (see project on the OSF for a full list of measures and verbatim items).

#### Main Dependent Variables

As in Experiment 1, participants’ weight loss (i.e., difference in weight_T1_ − weight_T2_) and the goal discrepancy (difference of desired minus actual weight loss; in %) served as our key dependent variables.

### Results

#### Preliminary Analyses (Preregistered)

The three experimental conditions did not differ in terms of starting weight (*M*_precise_ = 71.90 kg, *SD* = 12.74; *M*_round_ = 73.47 kg, *SD* = 13.17; *M*_control_ = 72.17 kg, *SD* = 13.07), *F*_weight_ (2, 147) = 0.21, *p* = 0.812, *η_p_*^2^ < 0.01. The ambitiousness of the self-set goals was again slightly lower for the precise than the round group (*M*_precise_ = 3.79 kg, *SD* = 1.59; *M*_round_ = 4.31 kg, *SD* = 1.92) but this descriptive difference was not significant as in Study 1, *t*(101) = 1.50, *p* = 0.136, *d* = 0.29 (please refer to the SOM for additional preliminary analyses). A Shapiro–Wilk test for our main dependent variables, weight loss and goal discrepancy, showed that both variables were normally distributed (*W*_weight loss_ = 0.99, *p* = 0.331; *W*_goal discrepancy_ = 0.99, *p* = 0.450).

#### Goal Setting Efficacy for Weight Loss (Preregistered)

**Figure 2:**
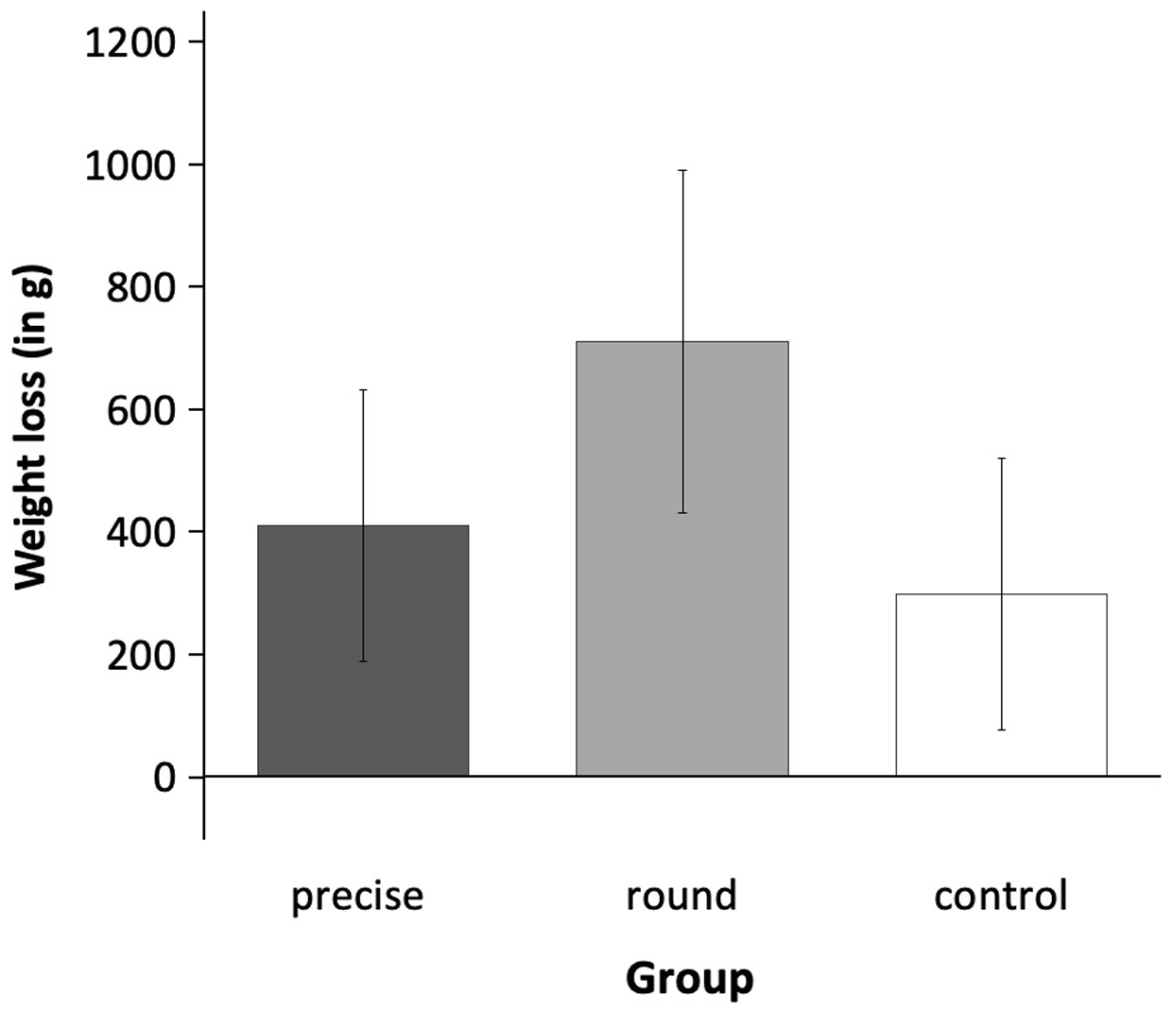
Weight loss in Experiment 2. The three experimental conditions (precise goal vs. round goal vs. control) did not differ significantly in weight loss. Error bars represent ±SEM.

A between-subjects ANOVA with weight loss as the dependent variable showed that the three groups did not differ significantly, *F*(2, 147) = 0.78, *p* = 0.459, *η_p_*^2^ = 0.2 (*M*_precise_ = 0.41 kg, *SD*_precise_ = 1.64; *M*_round_ = 0.71 kg, *SD*_round_ = 1.96; *M*_control_ = 0.30 kg, *SD*_control_ = 1.33; see Figure 2). *Post hoc* contrast analyses (−2 1 1) showed that, when considered jointly, the two goal groups did not lose significantly more weight than the control condition, *t*(147) = −0.88, *p* = 0.381, *d* = 0.16.

#### Goal Setting Efficacy for Weight Loss (Non-preregistered)

We calculated one-sample *t*-test in order to check whether the weight loss of each of the three groups differed significantly from zero, which was not the case for the control condition [*t*(46) = 1.54, *p* = 0.131, *d* = 0.07], and the precise condition [*t*(53) = 1.82, *p* = 0.074, *d* = 0.07] but was significant for the round condition [*t*(48) = 2.52, *p* = 0.015, *d* = 0.1].

#### Precise Versus Round Goal Efficacy (Preregistered)

Descriptively, the round group (*M* = 0.71 kg, *SD* = 1.96) lost more weight than the precise group (*M* = 0.41 kg, *SD* = 1.64), but this difference was not significant, *t*(101) = 0.85, *p* = 0.399, *d* = 0.26. We also checked how much participants’ actual weight loss differed from their desired weight loss (goal discrepancy). On average, the round group achieved their goal to 32.14%, while the precise group achieved their goal to 20.80%. Again, this difference was not significant, *t*(101) = 1.49, *p* = 0.139, *d* = 0.29.

#### Eating Behavior and Physical Exercise Behavior (Non-preregistered)

We also tested whether the three groups differed in their reported eating and physical exercise behavior. The results revealed that the three groups did not differ significantly in reported eating behavior, *F*(2, 147) = 2.21, *p* = 0.113, *η_p_*^2^ = 0.03, or in physical exercise behavior, *F*(2, 147) = 1.18, *p* = 0.309, *η_p_*^2^ = 0.02.

### Discussion

Contrary to the results of Experiment 1, Experiment 2 did not find significant weight loss differences between the three experimental groups. Descriptively, the round group lost the most weight, followed by the precise group and the control group. The goal groups did not differ in their reported eating and physical exercise behavior during the 8 weeks of the study.

## Internal Meta-Analytic Summary (Non-preregistered)

Given the inconsistent result patterns from both experiments, we conducted an internal meta-analytic summary—specifically, an “individual participant data” (IPD) meta-analysis (Burke et al., 2017). Multi-study papers that include studies with inconsistent findings profit from reporting internal meta-analyses that allow for more robust and cogent conclusions because the results are based on a larger sample with increased statistical power (Maner, 2014; Goh et al., 2016). Thus, internal meta-analyses deliver more reliable effect size estimates and are able to detect smaller effects that individual studies may be unable to detect. An IPD meta-analysis is based on the raw, participant-level data from each study and thus has several advantages over a traditional meta-analysis that typically uses aggregated data (Chalmers, 1993; Riley et al., 2010; Kaufmann et al., 2016). For example, IPD meta-analyses allow to investigate individual-level variables as potential mediators or moderators which is not possible in typical meta-analyses that use study-level information (Huh et al., 2015). Moreover, IPD meta-analyses increase statistical power to investigate potential mediators and moderators (Haasova et al., 2014; Breedvelt et al., 2020). We used the one-stage IPD approach by analyzing all data simultaneously while preserving the clustering within studies (Riley et al., 2020).

### Methods

Following common IPD procedures (e.g., Riley et al., 2020), we synthesized the data of each experiment into a single data set. As in the preregistered experiments, participants were again excluded when they were in a goal group and did not set themselves a goal, chose a goal that did not correspond to their experimental condition, when the software failed to record their data, or when their weight loss exceeded more than ±2.5 *SD* from the respective group mean. The synthesized data set included *N* = 270 participants. With this IPD, we compared the weight loss (weight_T1_ – weight_T2_) of the three groups while controlling for the study that the entered data stemmed from (study 1 vs. study 2). Thus, study served as a covariate to preserve the clustering of participants within the studies (Riley et al., 2010).

### Meta-Analytic Results

**Figure 3:**
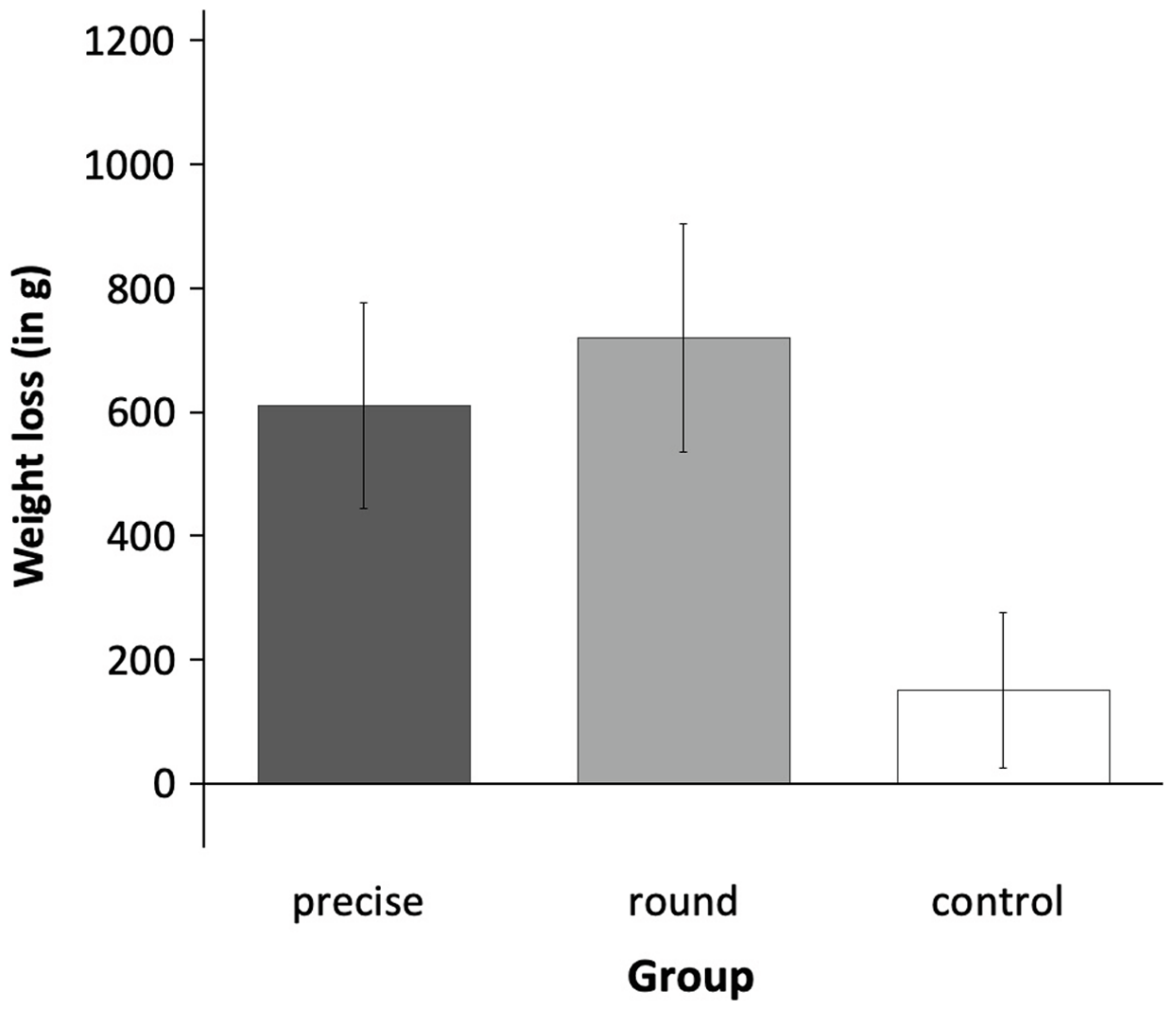
Weight loss across Experiment 1 and Experiment 2. The individual participant data (IPD) meta-analysis showed that across both experiments, the three experimental conditions (precise goal vs. round goal vs. control) differed significantly in weight loss. Contrast analyses showed that both the precise goal and the round goal condition lost significantly more weight than the waiting control group. Error bars represent ±1 SEM.

The IPD meta-analytical results showed a significant effect in that the three conditions differed in weight loss, *F*(2, 266) = 3.47, *p* = 0.033, *η_p_*^2^ = 0.03 (Figure 3). The covariate “study” was non-significant, *F*(1, 266) = 0.05, *p* = 0.819, *η_p_*^2^ < 0.01.

A planned *post hoc* contrast analysis (−2 +1 +1) showed that participants in the goal groups (coded as +1) lost significantly more weight than participants in the control group (coded −2; *M*_precise_ = 0.61 kg, *SD* = 1.66; *M*_round_ = 0.72 kg, *SD* = 1.66; *M*_control_ = 0.15 kg, *SD* = 1.18; Figure 3), *t*(267) = −2.61, *p* = 0.009, *d* = 0.32 (see H1).

The difference between the precise and the round goal group was non-significant, *t*(267) = 0.49, *p* = 0.625, *d* = 0.06 (see H2). The difference between the round goal and the control condition was significant, *t*(267) = −2.44, *p* = 0.015, *d* = 0.29, as was the difference between the precise goal and the control condition, *t*(267) = −2.10, *p* = 0.037, *d* = 0.25.

For the goal groups, we also tested how much participants’ actual weight loss differed from their desired weight loss (i.e., goal discrepancy). On average, individuals in the precise group achieved their goal to a percentage of 29.78%, while the round group achieved their goal to a percentage of 30.46%. This difference was not significant, *t*(181) = −0.12, *p* = 0.909, *d* = 0.02.

### Auxiliary Analyses

One major advantage of IPD meta-analysis is that it allows for the investigation of potential mediators on the level of IPD (Haasova et al., 2014). To obtain reasonable statistical power in mediation analyses assuming realistic effect sizes, fairly large sample sizes are necessary (e.g., Fritz and MacKinnon, 2007). For instance, in order to detect an indirect effect of small-to-medium size with a statistical power of 0.80, we need at least 162 participants with the efficient percentile bootstrapping method (see Fritz and MacKinnon, 2007; table 3, column HH). The smaller sample sizes in Experiment 1 (*N* = 121) and Experiment 2 (*N* = 150) were thus not sufficiently powered for mediation analyses. The meta-analytic IPD approach allowed us to investigate potential mediators with improved power.

#### Eating Behavior and Physical Exercise Behavior

**Figure 4:**
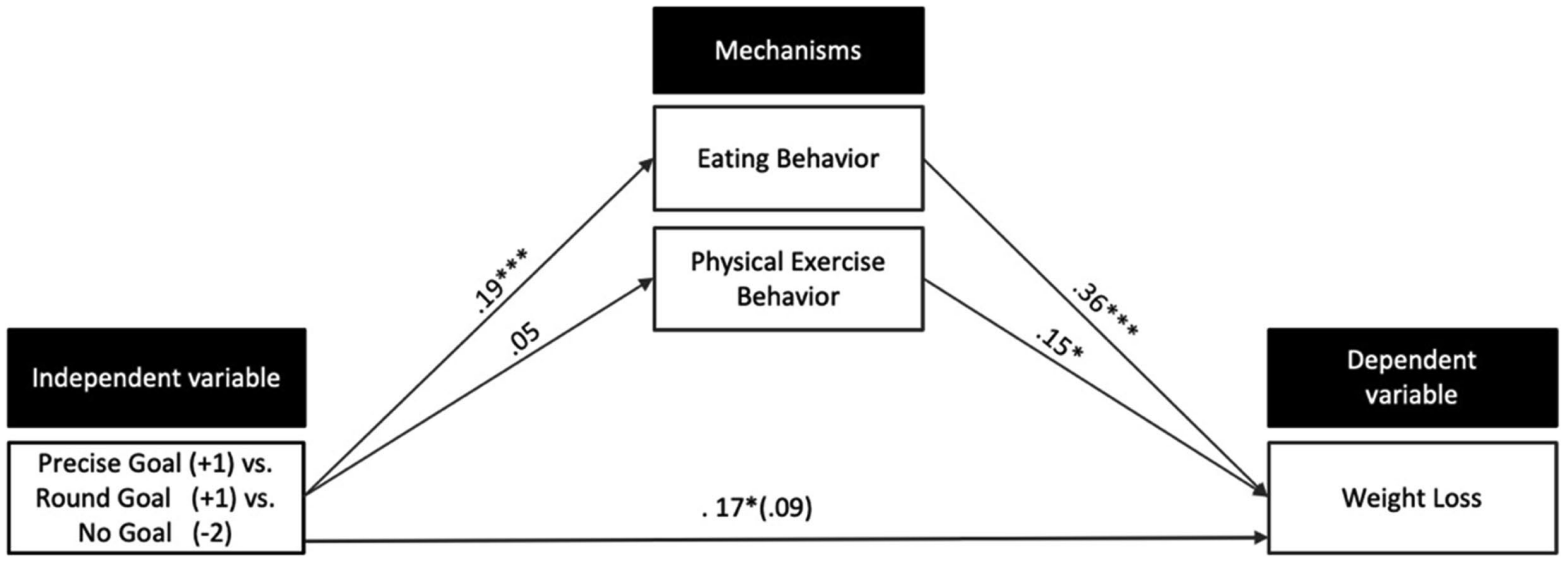
Multiple mediation model with IPD data across both experiments. The model shows that the effect of goals on weight loss was mediated *via* participants’ eating behavior during the weight loss phase. The indirect effect *via* physical exercise was non-significant. Path coefficients are standardized regression weights with values of *p*. ^*^p < 0.05; ^***^p < 0.001.

In both experiments, we also assessed participants’ self-reported eating behavior and physical exercise behavior because typically individuals try to lose weight by adopting a healthier eating behavior or by being physically active. We used multiple mediation analysis to test why across both experiments the goal conditions succeeded to lose more weight than the waiting control condition. We simultaneously tested for two indirect effects (eating behavior and physical exercise behavior) with 5,000 bootstrapping iterations (Process macro; Hayes, 2013; Model 4). The three experimental conditions (coded: +1 = precise, +1 = round, −2 = control) served as the independent variable, weight loss difference (time 1 − time 2) as the dependent variable. Results revealed that the indirect effect through eating behavior was significant [*b* = 0.069, SE = 0.023, CI_95%_ (+0.030; +0.123); zero is not included in the CI], while the indirect effect through physical exercise behavior was non-significant [*b* = 0.007, SE = 0.010, CI_95%_ (−0.009; 0.031); see Figure 4].

### Discussion

The internal IPD meta-analysis revealed that across both experiments, the goal conditions lost more weight than the control condition, while there was no significant difference between the precise and the round goal group. The greater power of the increased sample size of the IPD also allowed us to run auxiliary mediation analysis which showed that the more pronounced weight loss of the two goal groups was linked to a healthier eating (but not physical exercise) behavior during the weight loss phase.

## General Discussion

Research and theorizing on numeric precision effects allow for competing predictions as to whether precise or round goals are more effective for weight loss. The present research expands the goal setting literature by examining goal precision as a novel goal characteristic and by contrasting the competing hypotheses of the scale-granularity account and the reference point account. Experiment 1 showed that when considered jointly both goal groups lost more weight compared to the waiting control group. The precise goal group lost slightly more weight and approached their targeted goals more closely than the round goal group, but these descriptive differences were not significant. Experiment 2 sought to replicate these findings, but did not find significant differences in weight loss between the three groups. An IPD meta-analysis across both experiments allowed us to draw more robust and cogent conclusions across data from both studies. The IPD approach also has advantages over classical meta-analyses, such as the possibility to examine potential mediators and moderators on individual-level information and increased statistical power (Huh et al., 2015; Breedvelt et al., 2020). With respect to numeric precision, the IPD meta-analysis did not show differences between precise and round goals. Referring to the competing predictions as to whether round or precise goals are more effective, our data suggest that both goals are equally effective. Overall, the results of the IPD showed that both the precise and the round goal group lost significantly more weight than the control group. A multiple mediation analysis showed that the greater weight loss of the goal groups was linked to a self-reported healthier eating behavior.

In the following, we wish to discuss a number of potential reasons that could explain the lack of difference in precise versus round goals. First, this difference between precise versus round goals for weight loss success may simply not exist. Second, if we were to assume that there is a true effect, this effect may be smaller in size than expected, suggesting a lack of power. Third, we initially assumed that the longer time period of the weight loss phase in Experiment 2 (8 weeks instead of 6 weeks) could help to enhance the effect that goals exert on weight loss: the more time, the more weight could be lost, and the difference between experimental conditions might become more visible. Contrary to this assumption, however, the longer time period was not beneficial to reinforce the effect of precise versus round goals on weight loss: Indeed, at the end of appointment 2, we randomly chose 25 participants and asked them to draw a motivation curve that displays how their motivation to lose weight changed over the 8 weeks of weight loss. The majority of participants stated that their motivation tremendously decreased after 5 weeks of the experiment (*n* = 16, 64%). Participants, who were mainly students (*n* = 145), also reported that the last 2 weeks of weight loss coincided with their examination weeks that had begun at the end of the semester. Therefore, participants reported to have paid much less attention to their weight loss, eating behavior, and engaged less often in physical exercise compared to the beginning of the experiment. Thus, potential differences in weight loss between the three groups may have been attenuated (rather than fostered) due to the longer duration of Experiment 2. We can draw a two-fold conclusion: First, it seems that the manipulation was not strong enough to last over the entire time period of 8 weeks. Second, it seems plausible that the overlap of the experiment with students’ examination phase may have inadvertently decreased their motivation after week 5.

### Future Research

From Experiment 2, some factors can be derived that need to be considered in future studies. First, the time period for weight loss has to be chosen carefully. To avoid a decrease in motivation over time, future studies should aim for a stronger, continuous, and possibly self-reinforcing manipulation. For example, participants could be invited into the laboratory for an additional appointment after half of the weight loss period in order to take body measurements and to remind them of their goal. Participants’ motivation, eating, and physical exercise behavior could be assessed continuously to attain a better understanding of the mediating process over time. Future studies could also use experience sampling method (ESM) to gather information about individuals’ daily experiences and to realize a stronger study identification. ESM is an intensive longitudinal research procedure that allows signaling participants on multiple occasions over time and to ask them to report their thoughts, feelings, and behavior (Larson and Csikszentmihalyi, 2014). For example, participants in a weight loss study could be asked to report their weight on a daily basis, as well as their daily eating/physical exercise behavior, and their motivation to lose weight in order to attain a more detailed insight into participants’ (psychological) processes of losing weight effectively. Investigating participants daily behavior and motivation on different, more sensitive levels (e.g., daily number of steps and caloric intake) could also provide valuable insights about the influence of goal precision on behavior. In line with the scale-granularity account, it might be the case that precise goals lead to more specific representations of behavior compared to round goals.

Future studies might also investigate numeric goal precision over an even longer period of time. Research on the promotion of habit behavior shows that the implementation of a new behavior—such as eating healthy or engaging in more physical exercise (with the ultimate aim to lose weight)—is not a linear process over time (Walter, 2018). There are certain critical moments in which the new behavior that should be implemented (e.g., physical exercise and healthy nutrition) is carried out less frequently. For example, in a study investigating the development of health behavior, participants who sought to change their eating or physical exercise behavior had a small setback between week 4 and week 5 in that they performed a new behavior less frequently (Walter, 2018). Applied to our research, it might be the case that the two goal manipulations do differ significantly, but that this difference does not develop linearly over time. As we realized only one measurement at the very end of our experiments, we do not know how possible motivational, emotional, and behavioral differences between the three experimental conditions may have changed over the course of the experiment. The aforementioned ESM could be used to signal participants at fixed times throughout the week to create self-reinforcing habits and to continuously ask them to report their weight, motivation, and behavior. Thus, possible fluctuations and differences in weight loss over time could be tracked in a more fine-grained measurement approach.

Regarding our waiting control group, one might argue that participants in this condition were not yet trying to lose weight because they learned that for them the experiment would start 6 weeks later. In contrast, however, the assumption that the control group did try to lose weight was corroborated empirically by the weight loss of this group. This assumption is further supported by the fact that we only recruited participants seeking to reduce their weight and therefore signing up to this experiment. Nevertheless, in future studies, the control group might be explicitly advised to set a “do your best” goal (rather than a numerical goal) in order to realize a more conservative comparison with the goal groups.

Our research indicates that setting goals (whether they are round or precise) leads to more weight loss success than setting no goal. These findings are in line with goal setting theory and are also consistent with previous weight loss studies showing that goal setting is an important strategy for effective behavior change (Shilts et al., 2004). According to Locke and Latham (2002), setting goals is applicable to any self-regulated activity. Thus, our results might also be applicable to other domains. For example, it might be reasonable to assume that people who set themselves a goal for the number of baskets they want to shoot when playing basketball are more successful than people who simply try to shoot as many baskets as possible. Similarly, people who set themselves a saving goal, that is, an amount of money they want to save, might be more successful compared to people who just try to save as much money as possible. Future studies are needed to further investigate the applicability of goal setting in different domains.

## Concluding Comments

The present longitudinal field experiments investigated the effectiveness of self-set weight loss goals and two competing theoretical accounts for how numeric goal precision could impact goal achievement. While Experiment 1 alluded to a potential advantage of precise weight loss goals, Experiment 2 did not replicate this pattern of results. Across both experiments, a meta-analysis showed that both goal groups lost significantly more weight than the waiting control group; however, the two goal groups did not differ significantly in weight loss. A multiple mediation analysis across both experiments revealed that the greater weight loss of the goal groups was due to a healthier eating behavior rather than physical exercise. Future research is needed to further investigate numeric precision as a novel goal characteristic.

## Data Availability Statement

The datasets presented in these studies can be found in online repositories. The names of the repository/repositories and accession number(s) can be found at: https://osf.io/mdkhx/; https://osf.io/aj78d/.

## Ethics Statement

Ethical review and approval were not required for the studies on human participants in accordance with the local legislation and institutional requirements. The ethics board of the Faculty of Business and Economics, Leuphana University Lüneburg and the Research Commission of the Faculty of Business and Economics provided general approval and confirmed that the present research is exempt from ethical review. All data was collected and analyzed anonymously. The participants provided their written informed consent to participate in these studies. Participants received a written debriefing at the end of each study.

## Author Contributions

M-LF, MF, and DL developed the research questions and study design. M-LF organized the database, performed the statistical analyses, and wrote the first draft of the manuscript. MF and DL provided critical revisions. All authors contributed to the article and approved the final version of the manuscript for submission.

## Funding

The research was supported by a grant from the German Research Foundation (DFG LO 2201/2-1) which was awarded to DL and MF.

## Conflict of Interest

The authors declare that the research was conducted in the absence of any commercial or financial relationships that could be construed as a potential conflict of interest.

## Publisher’s Note

All claims expressed in this article are solely those of the authors and do not necessarily represent those of their affiliated organizations, or those of the publisher, the editors and the reviewers. Any product that may be evaluated in this article, or claim that may be made by its manufacturer, is not guaranteed or endorsed by the publisher.

## Footnotes

^1^ https://osf.io/mdkhx/
