# Supplementary material for: “My Goal Is to Lose 2.923 kg!”—Efficacy of Precise Versus Round Goals for Body Weight Reduction
Source: Front Psychol. 2022 Feb 7;13:793962. doi: 10.3389/fpsyg.2022.793962 (PMC8860075; doi:10.3389/fpsyg.2022.793962)
Supplement: Supplementary file 1 [file Data_Sheet_1.pdf]

Additional Analyses

Preliminary Analyses. Participants in the three groups (precise vs. round vs. control) did not differ significantly in their self-control ($F[2, 118] = 0.38$, $p = .686$, $\eta_p^2 = 0.01$) and their weight efficacy ($F[2,118] = 2.12$, $p = .124$, $\eta_p^2 = 0.04$). However, participants of the three groups differed significantly in their self-efficacy for sports-related activities ($F[2, 118] = 3.24$, $p = .043$, $\eta_p^2 = 0.05$), in that the round group had a significantly lower self-efficacy ($M = 4.24$, $SD = 0.99$) for sports-related activities compared to the precise goal group ($M = 4.84$, $SD = 1.00$; $t[117]= -2.45$, $p = .016$, $d = 0.45$) and compared to the control group ($M = 4.74$, $SD = 1.16$; $t[117]= 2.00$, $p = .048$, $d = 0.37$). There is also a trend that participants differ in their restraint eating ($F[2, 118] = 3.03$, $p = .052$, $\eta_p^2 = 0.05$) indicating that the precise group ($M = 26.71$, $SD = 4.66$) has higher scores on the restraint eating scale than the control group ($M = 24.54$, $SD = 4.20$) and the round group ($M = 24.78$, $SD = 4.74$). Higher scores suggest a higher likelihood of maladaptive eating attitudes and behaviors.

Goal Efficacy for other Body Measurements. We also checked whether the three groups differed in their reduction of BMI, body fat, visceral fat and in the increase of muscles mass. For our analyses we used the difference of each of these measures from appointment 1 and appointment 2 (i.e., difference $BMI_{T1} – BMI_{T2}$).

Participants of the three groups did not differ significantly in BMI ($F[2,118] = 2.04$, $p = .135$, $\eta_p^2 = 0.03$), body fat ($F[2,118] = 0.47$, $p = .624$, $\eta_p^2 = 0.01$), and visceral fat reduction ($F[2,118] = 0.54$, $p = .582$, $\eta_p^2 < 0.01$). Neither they differed in the increase of muscle mass ($F[2,118] = 0.24$, $p = .785$, $\eta_p^2 < 0.01$).

Robustness Checks. We also calculated separate ANCOVAs that controlled for age, gender, Weight Watcher vs. student participants, self-control, weight-efficacy, self-efficacy for sports-related activities and restraint eating. These analyses constantly showed the same effect of the experimental conditions on weight loss; $F_{age}(2,117) = 3.80$, $p = .025$, $\eta_p^2 = 0.06$;

$F_{gender}(2,117) = 3.82$, $p = .025$, $\eta_p^2 = 0.06$; $F_{Weight\ Watchers}(2,117) = 3.74$, $p = .027$, $\eta_p^2 = 0.06$), $F_{self\text{-}control}(2,117) = 4.38$, $p = .015$, $\eta_p^2 = 0.07$, $F_{weight\text{-}efficacy}(2,117) = 4.23$, $p = .017$, $\eta_p^2 = 0.07$, $F_{sports\text{-}related\ self\text{-}efficacy}(2,117) = 4.20$, $p = .017$, $\eta_p^2 = 0.07$, $F_{restraint\ eating}(2,117) = 3.22$, $p = .044$, $\eta_p^2 = 0.05$.

Goal Perception. We also checked whether precise goals lead to a finer scale resolution (see scale-granularity account; e.g., “I felt that I moved in small, continuous steps towards my goal”, eight items, Cronbach’s alpha = 0.79) and whether round goals served as motivating reference points (see reference-point account; “Once, I came closer to attaining my goal, I was particularly motivated to reach it”, seven items, Cronbach’s alpha = 0.73). Results showed that the two groups did not differ in their mental scale resolution ($M_{precise} = 3.91$, $SD_{precise} = 1.26$; $M_{round} = 3.85$, $SD_{round} = 1.09$), $t_{scale\text{-}granularity}(78) = -0.21$, $p = .831$, $d = 0.24$, or their perception of goals as reference points ($M_{precise} = 4.01$, $SD_{precise} = 1.08$; $M_{round} = 4.02$, $SD_{round} = 0.99$), $t_{reference\text{-}point}(78) = 0.02$, $p = .980$, $d = 0.24$.

We also tested how difficult participants perceived their goal (e.g., “My goal frustrated me”, “My goal has challenged me”, six items, Cronbach’s alpha = 0.61) and whether participants felt a personal relation to their goal (i.e., “I always had my goal in mind”, “I have a personal connection to my goal”, two items, Cronbach’s alpha = 0.68)[1]. Results showed that there is no difference between the precise group ($M_{difficulty} = 3.98$, $SD = 1.07$, $M_{personal\text{-}relation} = 5.23$, $SD = 1.54$) and the round group ($M_{difficulty} = 4.23$, $SD = 1.05$, $M_{personal\text{-}relation} = 5.38$ $SD = 1.18$), $t_{difficulty}(78) = 1.01$, $p = .318$, $d = 0.24$, $t_{personal\text{-}relation}(78) = 0.45$, $p = .652$, $d = 0.24$.

[1] For the items of each of the four scales, the scree plot of exploratory factor analyses indicated a one-factor solution.
