# Supplementary material for: “My Goal Is to Lose 2.923 kg!”—Efficacy of Precise Versus Round Goals for Body Weight Reduction
Source: Front Psychol. 2022 Feb 7;13:793962. doi: 10.3389/fpsyg.2022.793962 (PMC8860075; doi:10.3389/fpsyg.2022.793962)
Supplement: Supplementary file 2 [file Data_Sheet_2.pdf]

Additional Analyses

Preliminary Analyses. Participants in the three groups (precise vs. round vs. control) did not differ significantly in their self-control (F[2, 147] = 0.51, p = .600, η_p^2 = 0.01), their weight efficacy (F[2,147] = 1.04, p = .357, η_p^2 = 0.01), their self-efficacy for sports-related activities (F[2, 147] = 1.70, p = .187, η_p^2 = 0.02) and their restraint eating (F[2, 147] = 0.59, p = .557, η_p^2 = 0.01).

Goal Efficiency for other Body Measurements. We also checked whether the three groups differed in their reduction of BMI, body fat, visceral fat and in the increase of muscles mass. For our analyses we used the difference of each of these measures from appointment 1 and appointment 2 (i.e., difference $BMI_{T1} - BMI_{T2}$).

Participants of the three groups did not differ significantly in BMI (F[2,147] = 0.20, p = .819, $\eta_p^2 < 0.01$), body fat (F[2,147] = 0.49, p = .613, η_p^2 = 0.01), and visceral fat reduction (F[2,147] = 1.18, p = .311, η_p^2 = 0.01). Neither they differed in the increase of muscle mass (F[2,147] = 0.29, p = .748, η_p^2 = 0.00).

Goal Perception. We also checked whether precise goals lead to a finer scale resolution (see scale-granularity account; e.g., "I felt that I moved in small, continuous steps towards my goal", eight items, Cronbach's alpha = 0.80) and whether round goals served as motivating reference points (see reference-point account; "Once, I came closer to attaining my goal, I was particularly motivated to reach it", seven items, Cronbach's alpha = 0.73). Results showed that the two groups did not differ in their mental scale resolution ($M_{precise}$ = 3.15, $SD_{precise}$ = 1.03; M_{round} = 2.95, SD_{round} = 1.12), $t_{scale\text{-}granularity}(100)$= -0.96, p = .341, d = 0.23, or their perception of goals as reference points ($M_{precise}$ = 4.01, $SD_{precise}$ = 1.02; M_{round} = 2.95, SD_{round} = 1.12), $t_{reference\text{-}point}(100)$= -1.25, p = .214, d = 0.25). Additionally, six items tested how difficult participants perceived their goal (Cronbach's alpha = 0.71) and eight

items whether participants felt a personal relation to their goal (Cronbach's alpha = 0.82)[1]. Results showed that there is no difference between the precise ($M_{difficulty}$ = 4.39, SD = 1.23, $M_{personal\text{-}relation}$ = 4.50, SD = 1.34) and the round group ($M_{difficulty}$ = 4.11, SD = 1.36, $M_{personal\text{-}relation}$ = 4.60 SD = 1.05), $t_{difficulty}(100)$= -1.09, p = .282, d = 0.22, $t_{personal\text{-}relation}(100)$= 0.43, p = .671, d = 0.08.

[1] For the items of each of the four scales, the scree plot of exploratory factor analyses indicated a one-factor solution.
